# Mature retinal pigment epithelium cells are retained in the cell cycle and proliferate in vivo

**Published:** 2008-10-06

**Authors:** Heba Al-Hussaini, Jaimie Hoh Kam, Anthony Vugler, Ma’ayan Semo, Glen Jeffery

**Affiliations:** Institute of Ophthalmology, University College London, United Kingdom

## Abstract

**Purpose:**

To investigate the capacity of mature retinal pigment epithelium (RPE) cells to enter the cell cycle in vivo using a range of RPE-specific and proliferative specific markers in both pigmented and albino rats.

**Methods:**

Whole-mounted retinas of both Dark Agouti and albino rats were immunolabeled with cell cycle markers Ki67 or PCNA and double labeled with RPE cell marker RPE65 or CRALBP. The number and distribution of these cells was mapped. An additional group of Dark Agouti rats were given repeated intraperitoneal injections of Bromodeoxyuridine (BrdU )for 20 days and then sacrificed 30 days later. The retinas were then processed for BrdU detection and Otx, a RPE cell-specific marker. For comparison, human RPE tissue from a postmortem donor was also labeled for Ki67.

**Results:**

In both pigmentation phenotypes, a subpopulation of mature RPE cells in the periphery were positive for both cell cycle markers. These cells were negative for Caspase 3, hence were not apoptotic. Ki67-positive cells were also seen in human RPE. Further, many cells positive for BrdU were identified in similar retinal regions, confirming that RPE cells not only enter the cell cycle but also divide, albeit at a slow cell cycle rate. There was a ten fold increase in the number of RPE cells positive for cell cycle markers in albino (approximately 200 cells) compared to pigmented rats (approximately 20 cells).

**Conclusions:**

Peripheral RPE cells in rats have the capacity to enter the cell cycle and complete cellular division.

## Introduction

In the mature mammalian retina, cells such as neurons [1-4] and retinal pigment epithelium (RPE) [5,6] are lost with age. While there is no evidence of cell proliferation, an ocular stem cell population has been identified in the ciliary body [7]. Another ocular pigmented tissue that has the latent capacity to replicate is the RPE. The RPE is an integral part of the retina and plays a critical role in both neural retinal development and retinal function. The mature RPE is held in a state of senescence by the adjacent neural retina, because when the retina is detached, RPE cells proliferate [8]. In the amphibian, removal of the neural retina results in RPE cell proliferation as in mammals, but these cells then transdifferentiate to produce a completely new functional retina. In amphibians, the mature, but not the developing, RPE expresses the tissue specific marker RPE65. Following retinal removal RPE cells downregulate RPE65 while transdifferentiating, and it is only upregulated when retinal production is complete and transdifferentiation ceases [9]. Hence, it appears that, like the amphibian RPE, the mammalian RPE has the capacity to proliferate but not to transdifferentiate into the diverse cell types found in the retina.

Here we use three independent RPE cell markers, RPE65, Cellular retinaldehyde-binding protein (CRALBP), and Otx, making identification of these cells unambiguous. RPE65 is a key element in normal RPE function. It plays an important role in the visual cycle and in vitamin A metabolism. RPE65 is also associated with retinol binding protein and 11-cis-retinol dehydrogenase [10]. CRALBP binds to 11-cis-retinal in the visual cycle, and its function is associated with normal dark adaptation [11]. Otx2 is a specific marker for RPE cells an important in their specification [12]. There is an anecdotal evidence for cell cycle events in mature albino RPE [13]. Hence, here we examine the RPE in mature rats and human using three independent proliferative markers, Ki67, proliferating cell nuclear antigen (PCNA), and BrdU, to assess the latent capacity of this tissue to proliferate while the retina is in place. Both pigmented and albino rats are used, as during development albino retinas are abnormally proliferative due to the absence of L-dopa, a key cell cycle regulator and an upstream element in melanin synthesis [14,15].

## Methods

### Tissue source

Pairs of eyes were obtained from 2-month-old Dark Agouti (DA) rats (n=14) and albino Wistar rats (n=10) Pairs of eyes were also obtained from 1-year-old DA rats (n=5) and albino Wistar rats (n=5). DA rats were also taken at the following embryonic (E) and postnatal (P) days: E18 (n=3), P0 (n=5), P5 (n=4), P10 (n=5), P15 (n=6), P20 (n=5), P25 (n=5), and P45 (n=6). Four additional rats were taken at P90 and P150. Animals were euthanized by carbon dioxide inhalation. When rats were collected at E18, the mother was euthanized by carbon dioxide inhalation and the pups removed. The eyes from pups were enucleated and fixed in 4% paraformaldehyde overnight, and the anterior segment, vitreous, and retina removed leaving the eye cup with the RPE exposed. All procedures were performed under UK Government (Home Office) and local animal ethics committee approval.

An additional group of 18 P25 DA rats were given intraperitoneal injections of 50 µg/kg BrdU in 0.007 NaOH/phosphate buffered saline (PBS). This additional group was divided into six groups of three animals each. The first group of 3 was given a single injection and euthanized 3 h later. The second group of animals were injected at 3 h intervals and given a total of 4 injections before being euthanized. The third group was given a single injection and was euthanized 12 h later. The fourth group were given 4 injections separated by 12 h and euthanized 12 h after the last injection. The fifth group was given 5 injections separated by 24 h in a similar way and euthanized. The final group was injected once each day for 20 days and then euthanized one month after the last dose of BrdU. All animals were euthanized with carbon dioxide and the eyecups were fixed in 4% paraformaldehyde for 30 min. These experiments were undertaken to confirm that cell addition was taking place and cast limited light on the length of the cell cycle.

Healthy eyes from an 83-year-old postmortem donor were obtained from the Eye Bank at Moorfields Eye Hospital. Full Local Research Ethic Committee approval and appropriate consent were obtained under The Human Tissue Act. Consent is given prior to death or from the relatives following death. The eyes was approximately 36 h postmortem. The eyecups were fixed in 4% paraformaldehyde for 30 min, and the RPE and the underlying choroid removed as a single tissue sheet. Approximately one-third of this large sheet was trimmed to span the equatorial to peripheral retina so it could be used for analysis.

### Tissue staining

The rat eye cups containing the RPE were washed four times in 0.1 M PBS (pH 7.4), then blocked with 5% normal donkey serum (NDS) in 3% Triton X-100 in PBS for 2 h. Primary antibody incubation with 1:2,000 dilution of rabbit anti-Ki67 (Novocastra, Newcastle, UK) and 1:500 dilution of rabbit antiproliferative cell nuclear antigen (PCNA; Abcam, Cambridge, UK) in 1% NDS in 3% Triton X−100 in PBS was performed overnight at room temperature. Primary analysis was undertaken on tissue stained with Ki67, and PCNA was used in a confirmatory role. In most of the albino Wistar rats and approximately half of the DA rats, RPE cells were labeled with a second primary monoclonal antibody; we used a 1:500 dilution of either mouse anti-RPE65 (Chemicon, Hampshire, UK) or mouse anti-CRALBP (Affinity BioReagents, Cambridge, UK). Without this second monoclonal antibody, albino RPE was impossible to image. Following four washes in PBS the tissue was incubated for 2 h in a secondary antibody that consisted of a 1:200 dilution of TRITC donkey antimouse and FITC donkey antirabbit (Jackson ImmunoResearch laboratories, West Grove, PA) in 1% NDS in 0.3% Triton X-100. A 1:2,500 dilution of DAPI in PBS was added to the tissue for one min to label the nuclei of cells in the tissue. The eyecups were then washed extensively in 0.05 M Tris buffer (pH 7.4), mounted flat RPE up with Vecta shield, and examined under fluorescent microscopy. The same protocol of labeling was used for the RPE sheet taken from human tissue; however, only Ki67 and not RPE65 was used. As a negative control, some RPE cups were processed in the absence of primary antibodies.

Cell cycle proteins can be upregulated in mature cells when they initiate caspase-related apoptosis [16,17]. To control for this, we used caspase staining to determine if it would colocalize with cells positive for Ki67. For this we labeled with rabbit polyclonal anti-Caspase 3 (Abcam). Retinas were blocked as described in the last section and incubated overnight with a 1:500 dilution of Active Caspase 3 and 1:500 dilution of goat polyclonal anti-Ki67 (Santa Cruz Biotechnology, Santa Cruz, CA). Following four washes in PBS the tissue was incubated for 2 h in a 1:200 dilution of TRITC donkey antirabbit and FITC donkey antigoat (Jackson ImmunoResearch Laboratories,) in 1% NDS in 0.3% Triton X-100. DAPI was added, and the tissue was washed and flatmounted as described in the previous paragraph.

From the six rats injected with BrdU, one eye was processed for BrdU detection and the other one was double processed for BrdU detection and Ki67 or for BrdU and Otx. The eyes, which were double labeled, were first incubated overnight with either a 1:1,000 dilution of Ki67 or Otx (Santa Cruz Biotechnology) in 1% NDS in 3% Triton X-100 in PBS. The tissue was then incubated in the secondary antibody and fixed in 4% paraformaldehyde for 10 min.

Antigen retrieval was necessary for detection of BrdU in RPE cells. This was undertaken by placing the tissue in 6 M hydrochloric acid in 1% Triton X-100 in PBS for 30 min. Before incubation in BrdU the tissue was washed extensively with PBS to equilibrate the tissue to a normal pH. The tissue was then blocked with normal donkey serum for 2 h. An overnight incubation of 1:5 dilution of BrdU in 1% NDS in 3% Triton X-100 in PBS was performed at room temperature. Following four washes in PBS, the tissue was incubated for 2 h in a 1:200 dilution of TRITC donkey antimouse (Jackson ImmunoResearch Laboratories) in 1% NDS in 0.3% Triton X-100. The tissue was then washed once with PBS and extensively with Tris buffer, mounted flat, RPE up with vector shield, and examined under fluorescent microscope.

### Analysis

The number and distribution of Ki67-positive cells was mapped in the RPE flat mounts. The position of each cell was marked on a composite map, created using Adobe Photoshop CS version 8. RPE65 is a tissue-specific marker normally expressed in all RPE cells. Hence, only the variation in RPE65 expression was determined in cells positive for Ki67. When cells were positive for both Ki67 and RPE65 their position was plotted. Tissue processed for BrdU detection was mapped in a similar way with the relative location of BrdU and Ki67 plotted on schematic drawings. Human tissue was examined to see if Ki67 cells were detectable in the RPE. Because of the relatively large size of the human RPE tissue sheet it was necessary to process it in strips. This resulted in a loss of relative location of the positive cells, and consequently these were not plotted in relation to one another or other major land marks. Here, the primary aim was only to determine if they were present.

Many rodent RPE cells are binucleated [18]. To determine the relative distribution of these cells in relation to cell positive for cell cycle markers, we divided rodent RPE into three regions of approximately equivalent areas: central, equatorial, and peripheral. In each of these regions, three areas were analyzed measuring 150×150 μm. These were separated by approximately 120 degrees in relation to the optic nerve head, like three spokes of a wheel. Binucleated and mononucleated cells were counted within the defined areas. Cells with more than two nuclei were rarely encountered and were not recorded for the purpose of this study.

One-way ANOVA was used to analyze the statistical significance of Ki67 labeling in each experiment and was followed by post hoc Newman-Keuls multiple comparison test when appropriate.

## Results

**Figure 1:**
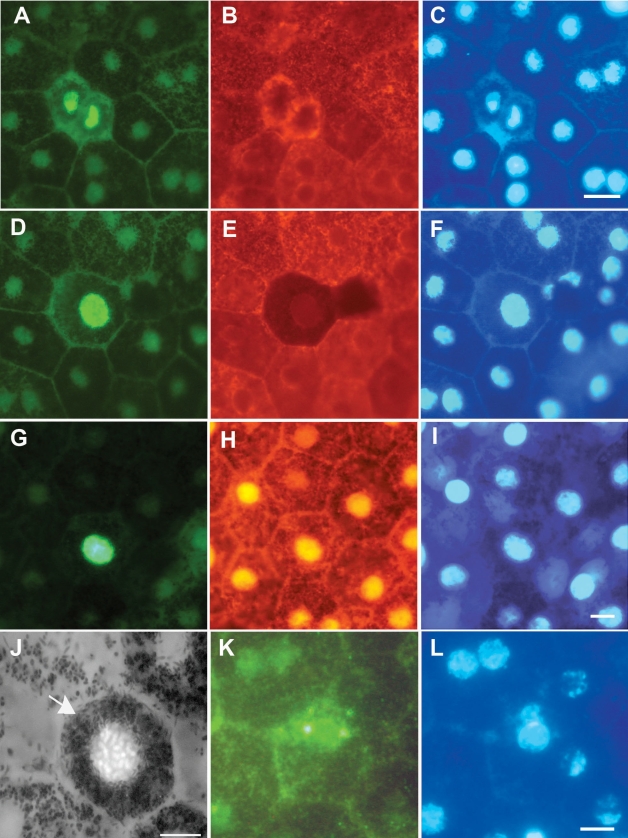
Labeling patterns in retinal pigment epithelium (RPE) sheets in whole mount preparations taken from DA rats. **A:** RPE cells positively labeled for Ki67. These appear to be in anaphase. **B:** This is the same region as shown in **A**, but stained with RPE65, which is an RPE specific marker in this tissue. **C:** This is the same region as shown in **A** and **B** but stained with DAPI to reveal the nuclei of the imaged cells. **D-F**: These are stained in the same way as **A-C**, however here the cell positive for Ki67 shown in **D** has down-regulated RPE65 as shown in **E**. **F** is the corresponding DAPI stained image. **G: **This shows a Ki67 positive RPE cell which has also in **H** been stained with CRALBP, which is a second RPE specific marker in this tissue. **I** is the same region stained with DAPI to reveal nuclei. **J** shows a Ki67 positive RPE cells. Taken in black and white the melanin granules in the cell can be clearly identified (arrow), which along with the RPE65 and CRALBP confirm that the tissue sheet examined is RPE. **K** shows an RPE cell positive for a second cell cell cycle marker PCNA, and **L** shows the same image stained with DAPI. The scale bar represents 10 µm.

RPE cells positively identified with RPE65 and CRALBP were apparent in all rat retinas examined. These cells had a clear hexagonal morphology. In the pigmented animals, all of the cells were packed with melanin granules. In both pigmented phenotypes, the size was consistent with RPE cells in a single hexagonal matrix in a single plane (Figure 1). Some of these cells were clearly labeled with the proliferative marker, Ki67 (Figure 1A,D); similar cells were also labeled with PCNA, the other proliferative marker used (Figure 1K,L). However, fewer cells were labeled with PCNA because Ki67 labels cells in all phases of the cell cycle except G_0_, while PCNA labels cells in S phase alone. Labeling with RPE65 and CRALBP clearly defined RPE cells in both pigmented (Figure 1) and albino rats (not shown), and confirmed that the cells in the cell cycle were truly RPE cells. However, some RPE cells that were Ki67 positive expressed low levels of RPE65, although the levels of expression were still greater than in negative controls (Figure 1D-F).

**Figure 2:**
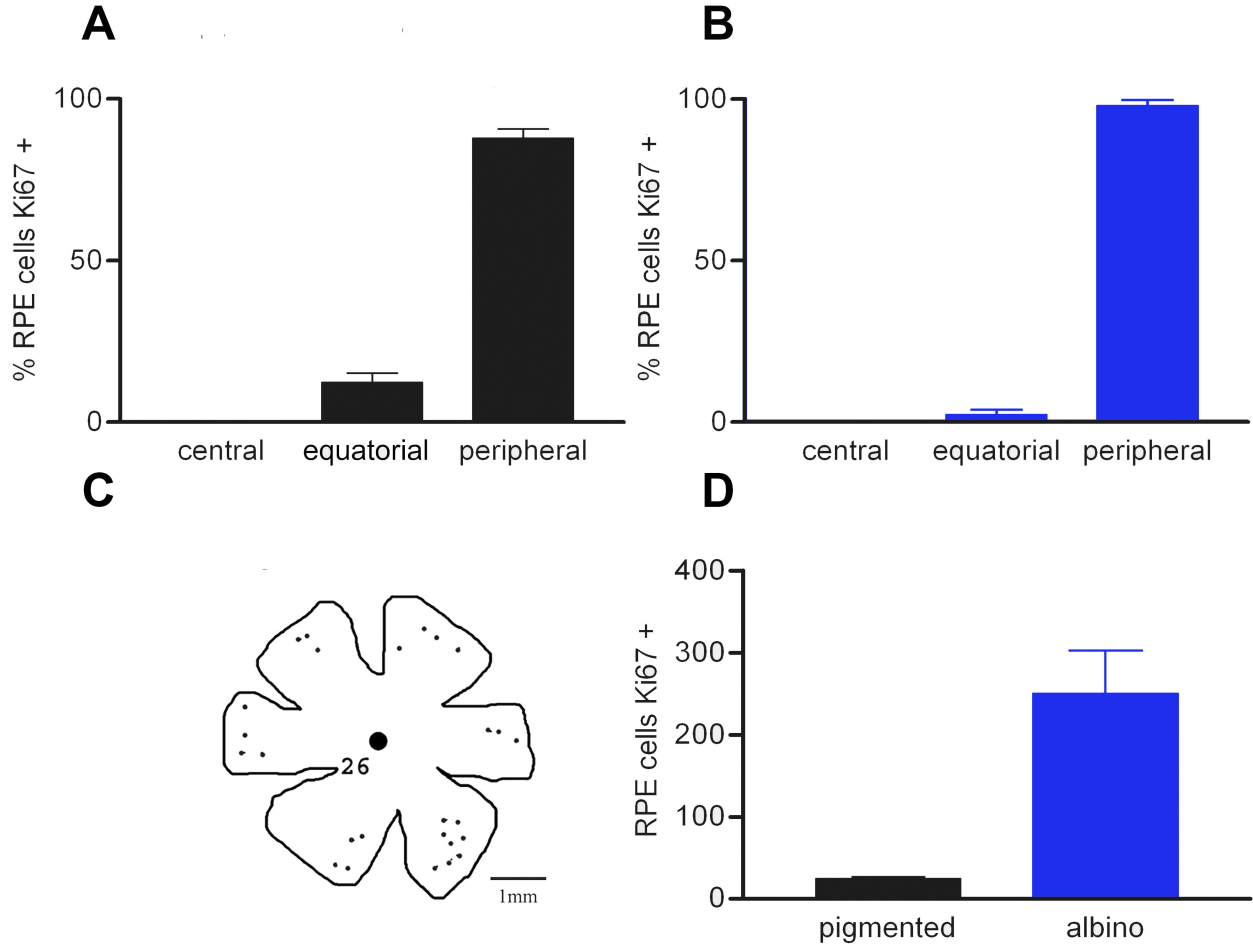
The distribution and number of Ki67 positive RPE cells in the retinae of pigmented (black bars) and albino (blue bars) rats. **A** and **B** show the relative distribution of Ki67 cells in retinae of pigmented and albino rats. In both cases the largest percentage of the total number of cells are located in the periphery with none in central retina regions. The actual distribution of these cells in a pigmented rat is given in **C**. **D** shows the absolute number of Ki67 positive RPE cells in a pigmented and an albino animals. The differences shown in **A** and **B** between the different retinal regions were statistically significant (ANOVA p<0.0001). Differences between equatorial and peripheral regions were also statistically significant (Newman Keules p<0.001). While the relative differences in the distribution of Ki67 cells within retinae was similar between pigmentation phenotypes, there were always many more RPE cells labeled with Ki67 in albinos compared with pigmented rats. This difference was statistically significant (Newman-Keuls p<0.01).

Ki67-labeled cells were only present in equatorial and peripheral regions (Figure 2). None were seen centrally, close to the optic nerve head. Negative controls in which all stages of immune processing were undertaken, except the application of the primary antibody, failed to show any label in the RPE in either central or peripheral locations with either Ki67 or PCNA. With the exception of cell numbers there were no geographic differences in the patterns of labeling found using either Ki67 or PCNA.

**Figure 3:**
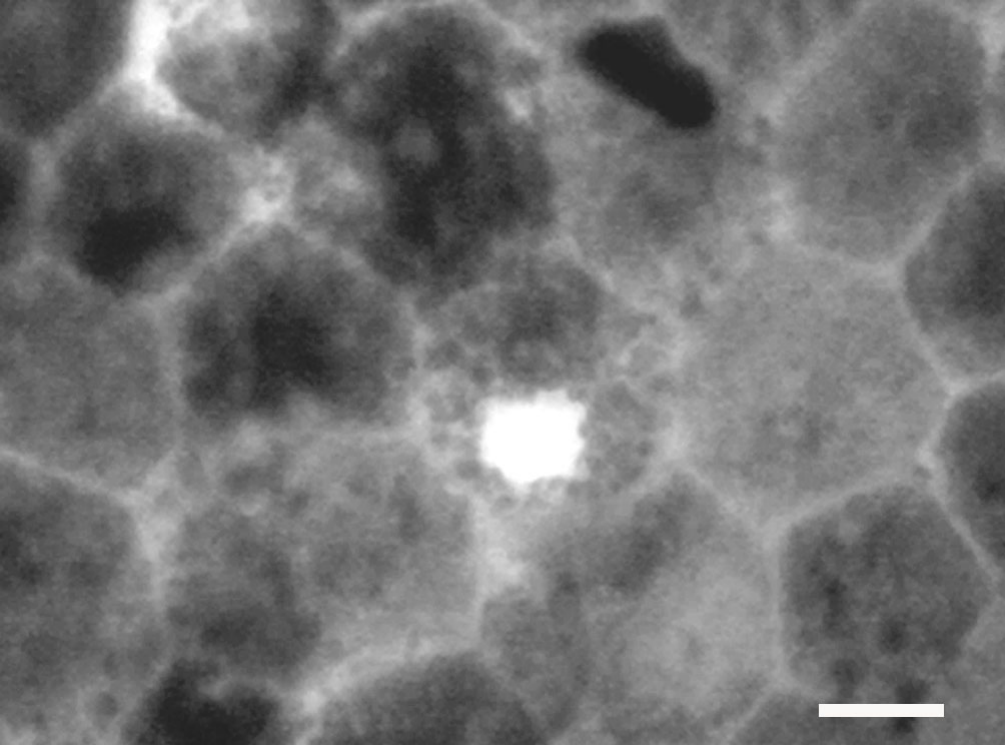
Ki67-positive cells in the sample of human eye tissue. Only a small strip of tissue, spanning from the equatorial to peripheral regions, was examined. While Ki67-positive cells were clearly present, it was not possible to estimate the number of these cells or map their retinal location. Scale bar equals 10 µm.

Because Ki67 labels a larger proportion of cells in the cell cycle, analysis focused on this proliferative marker. In 2-month-old pigmented rats the number of Ki67-positive cells found in the RPE was relatively small, being of the order of 20–30 in each retina (Figure 2C,D). Within the regions where they were found there was no obvious pattern in their distribution, other than their retinal location. The number of cells in the cell cycle in albino retinas was approximately ten fold greater than that found in pigmented animals (Figure 2D). In spite of this significant increase in their number, their distribution was similar to that found in pigmented animals (Figure 2A,B). In 1-year-old rats these numbers declined. In 1 year old DA rats these numbers declined to only 8-10 Ki67 positive cells, which was significantly less than found at 2 months of age (p<0.0001), and only around 15 in albinos, which again was a statistically significant age related reduction (p<0.0001). In spite of this decline, the numbers found in albino animals still remained significantly higher than in age-matched pigmented animals (p<0.01). The presence of Ki67-positive cells was also confirmed in human tissue (Figure 3), however, given that only a small sheet of human RPE was examined, it was only possible to confirm cell presence but not determine their number or distribution.

Cell cycle proteins can be upregulated in mature cells when they initiate Caspase related apoptosis [16,17]. To determine whether the Ki67-positive cells revealed here were dying, we stained for Ki67 and Caspase 3 in the same RPE tissue sheets. No Caspase 3 labeling was found in cells positive for Ki67. Hence, they were unlikely to be apoptotic.

**Figure 4:**
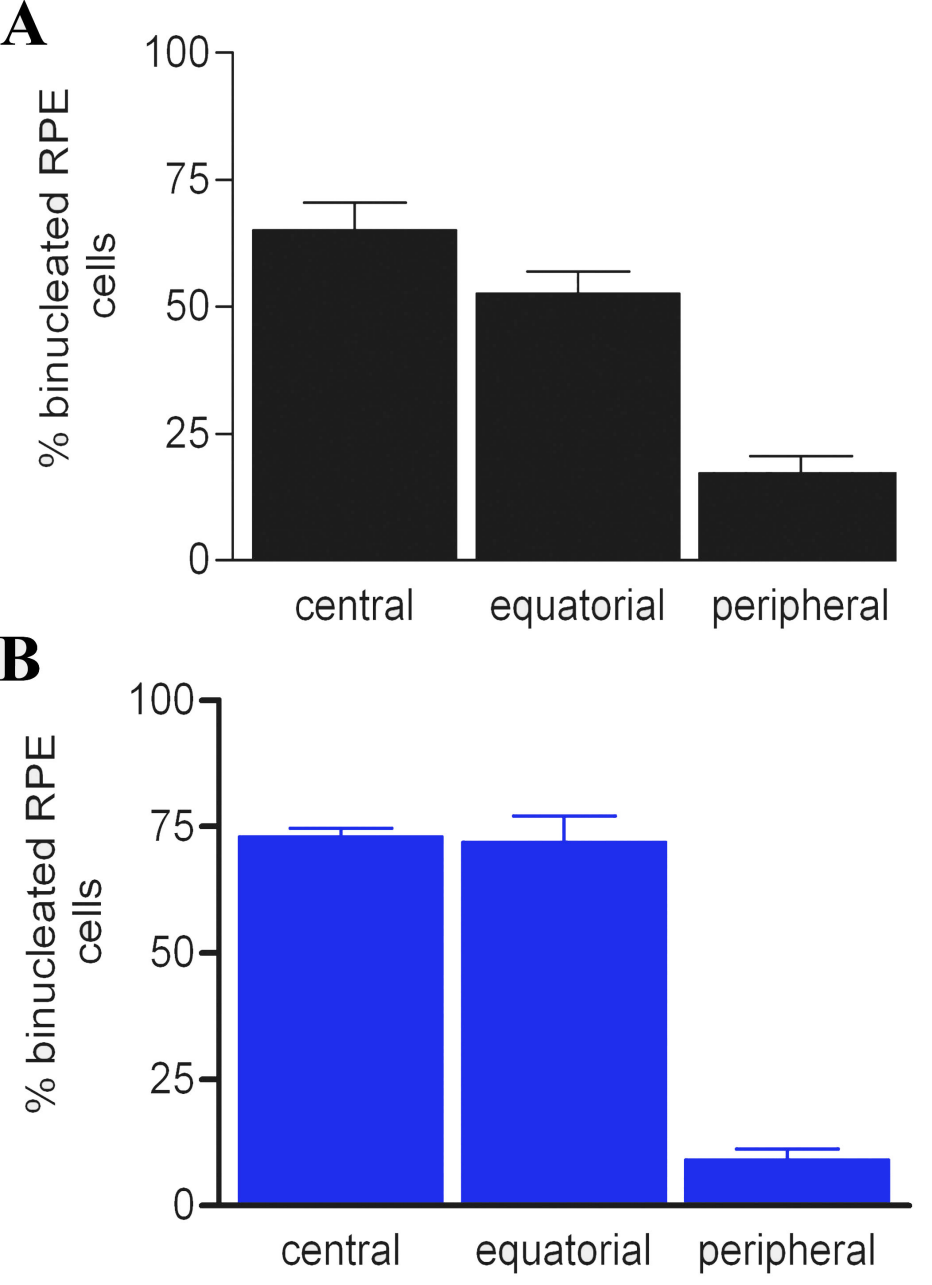
The percentage of binucleated RPE cells in different retinal regions in pigmented (black bars) and albino (blue bars) rats. In adult rodent retinas, many of the RPE cells were binucleated. The proportion of these have been determined in both pigmented (**A**) and albino (**B**) retinas. The retinas were divided into three roughly equal geographic regions: central, equatorial, and peripheral. In both pigmentation phenotypes the majority of the binucleated cells were located toward the central retina, although many were also found in equatorial regions. The distribution of these cells is the reverse pattern of that found for Ki67-positive cells shown in Figure 2. The differences in **A** and **B** are statistically significant (ANOVA, p<0.001). In both cases, the differences between the percentage of binucleated RPE cells between central and equatorial regions were not statistically significant. However, differences in the percentage of binucleated RPE cells found central and peripheral regions were significantly different (Newman-Keuls p<0.01). The differences between equatorial and peripheral regions were also statistically significant (Newman-Keuls p<0.01).

During the first two postnatal weeks, after cell production in the rodent RPE is largely complete, some RPE cells undergo nuclear division. This does not translate into full cell division, leaving a cellular population that is largely binucleated. The relative distribution of these cells has not been recorded [18]. Figure 4 shows the distribution of binucleated cells in pigmented and albino rat retinas that have been divided into central, equatorial, and peripheral regions. In both cases it is clear that the majority of binucleated cells are located in the central retina, with many spilling over into equatorial regions, but with relatively few in the periphery. RPE cells in central regions could also contain more than two nuclei. The small population of binucleated cells in the periphery were not obviously biased toward areas within this region adjacent to the equatorial sector. Hence, the distribution of binucleated cells was separate, although partly overlapping with that of the Ki67-positive cell population.

**Figure 5:**
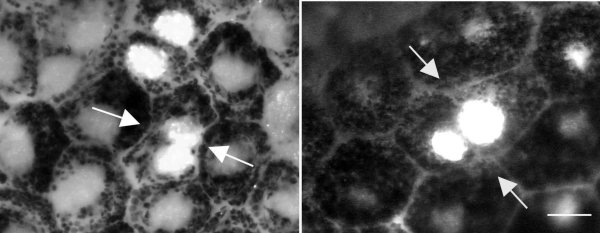
RPE cells were identified that appear to be going through full cell division. In the peripheral retinal regions, a small number of cells could be identified that appeared to be passing through full cell division. In both pictures, arrows point to two labeled nuclei that appear to be forming a plasma membrane between them. Both sets of cells appear irregular in the RPE cell matrix. Taken together with the finding that there was no increase in the number of binucleated cells in the peripheral retina, these photographs demonstrate that at least some of the cells in this region were undergoing full cell division.

**Figure 6:**
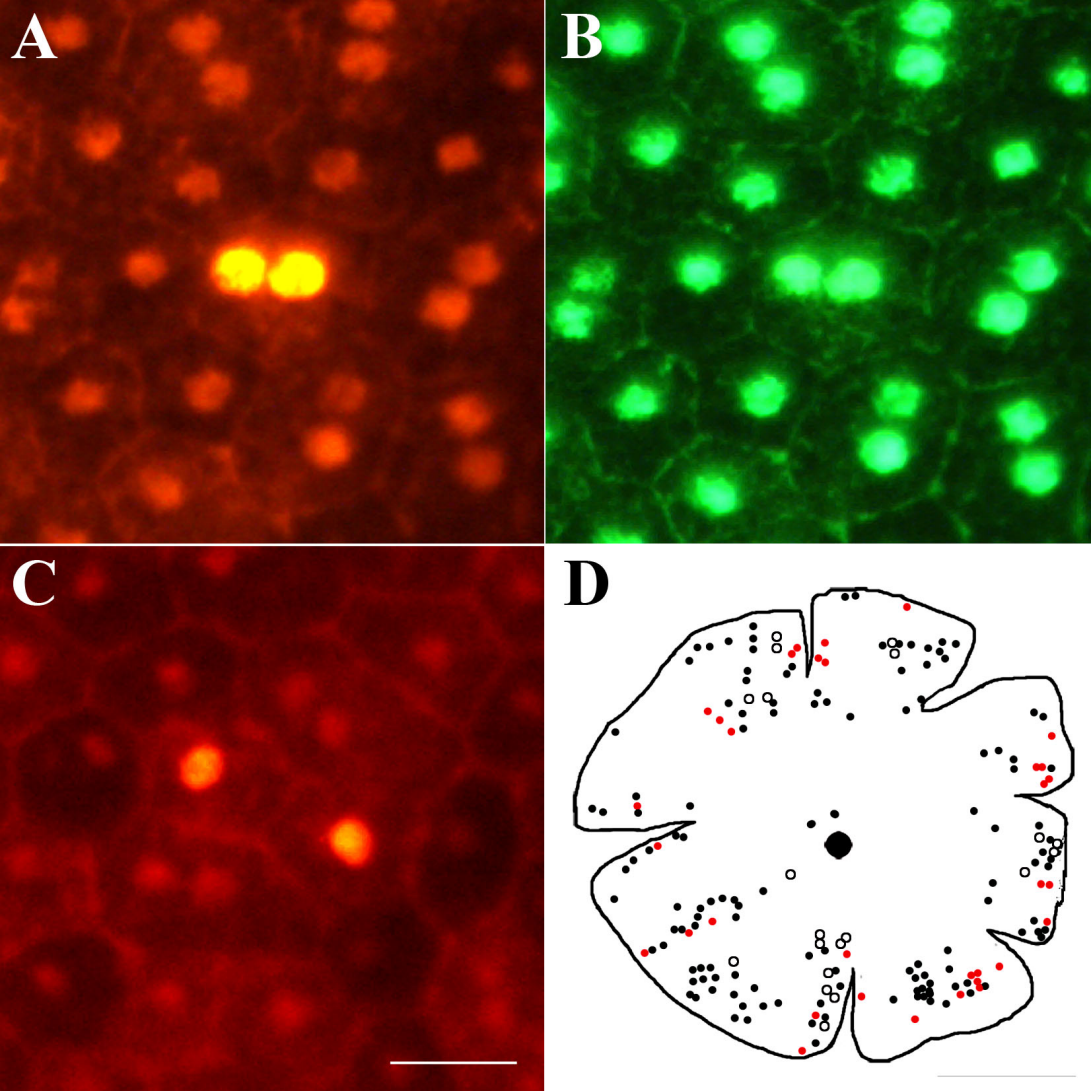
BrdU labeling in mature RPE cells. **A **and** B:** Shows a binucleated labeled cell with BrdU on red channel and Otx green channel. **C** shows two adjacent mono-nucleated cells that are labeled with BrdU. Scale bar equals 20 µm. **D** shows an outline drawing on which the location of RPE cells labeled with Ki67 and BrdU are marked. The diagram shows the distribution of positive BrdU-labeled binucleated (black dots) and mononucleated (black circle) cells. Only a small number of the BrdU-labeled cells were more centrally located than those labeled for Ki67. The mononucleated cells were almost always found in pairs of close proximity. The red dots represent the number and distribution of Ki67 positive RPE cells. While these largely overlap with the BrdU labeled population of RPE cells, they tend to occupy a slightly more peripheral location. The scale bar represents 2.5 mm.

**Figure 7:**
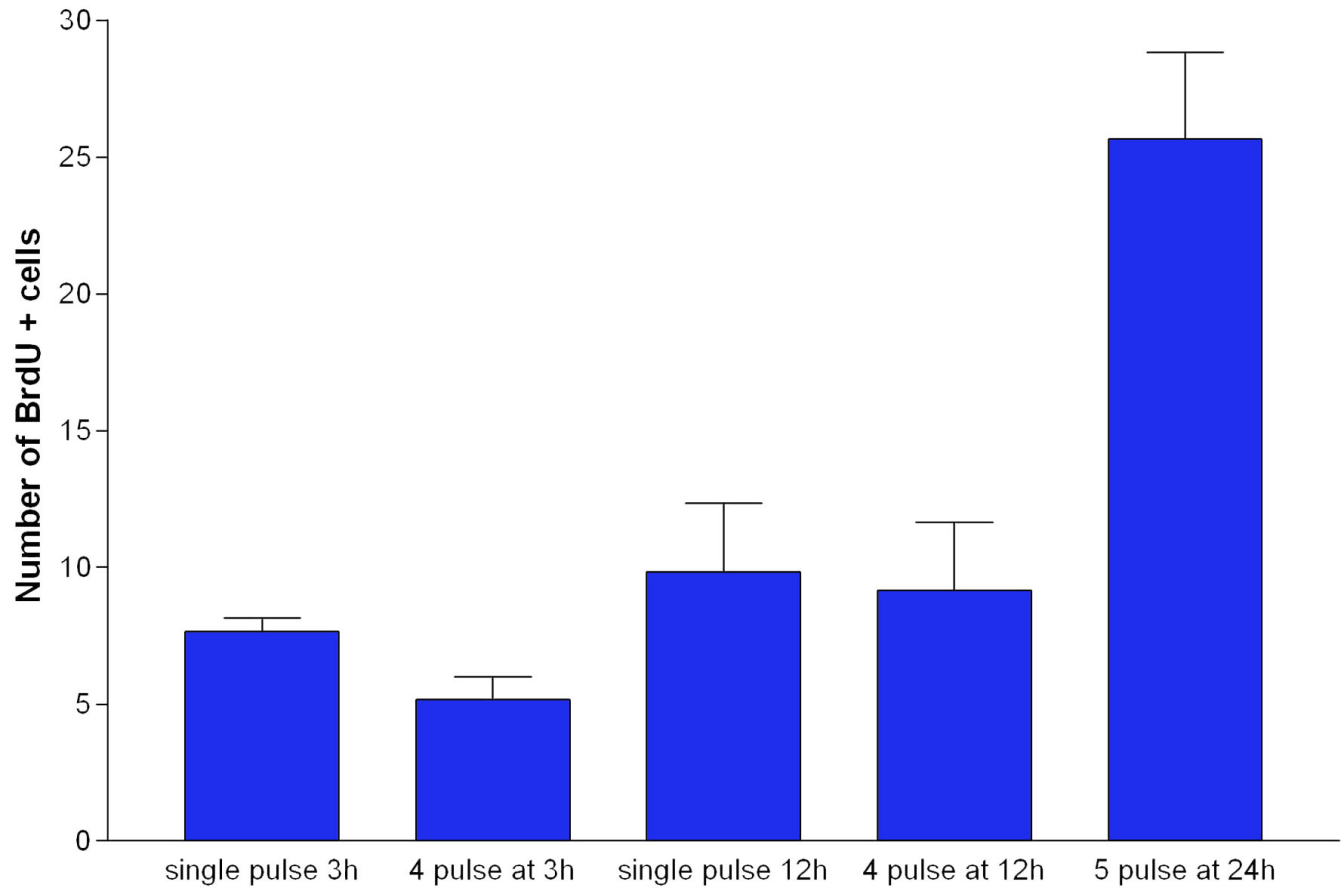
The number of BrdU positive cells in the RPE following multiple pulses at progressive times. BrdU was given as a single pulse (50µg/kg) or as multiple pulses in 2 month old DA rats. For comparison animals were given 4 injections at 3 h intervals and euthanized 3 h later. Similar experiments were undertaken at 12 h intervals, with single pulse and euthanized 12 h later compared with 4 pulses separated by 12 h each and euthanized 12 h after the last pulse. There was no statistically significant increase in labeled cells following multiple injections compared with single injections. However, when BrdU was pulsed at 24 h intervals over 5 days, there was a significant increase in the number of RPE labeled cells compared with labeling in any of the other groups (ANOVA, p<0.0001. Dunnett’s Multiple Comparison Test p<0.001). Hence the RPE population is proliferating, but with a cell cycle rate of approximately 5 days.

Are Ki67-positive cells in the periphery dividing or undergoing only nuclear division to become binucleated? Three lines of evidence support the notion that at least some of these cells are going through full cell division. First, if the nuclei of RPE cells are dividing but not the cell, then the number of binucleated cells should increase with age in the periphery. When the number of RPE binucleated cells at P20 and P60 were compared in the periphery, no significant difference was found in their number between the two ages (p>0.5). Second, there were cells identified that were Ki67 positive that possessed two labeled nuclei and appeared to be passing through full cell division, establishing a cytoplasmic membrane between their nuclei. These cells also appeared to be irregular within the geometric configuration of the regular RPE (Figure 5). Ki67 and PCNA are only associated with proliferation as they are simply cell cycle. However, the critical factor in favor of this resulting in cell division is that BrdU was detected in peripheral retinal cells. These were a mixed population with some having a single nucleus and others being binucleated. Cells with a single nucleus were commonly in adjacent pairs (Figure 6). Multiple injections of BrdU given at 3 and 12 h intervals failed to significantly increase the size of this population compared with animals given a single pulse. However, those given at 24 h intervals over 5 days did significantly expand the number of positive cells compared with all other groups (ANOVA=0.0001, Dunnett's Multiple Comparison Test=0.001, Figure 7). Hence, it is likely that the cell cycle rate is very slow.

**Figure 8:**
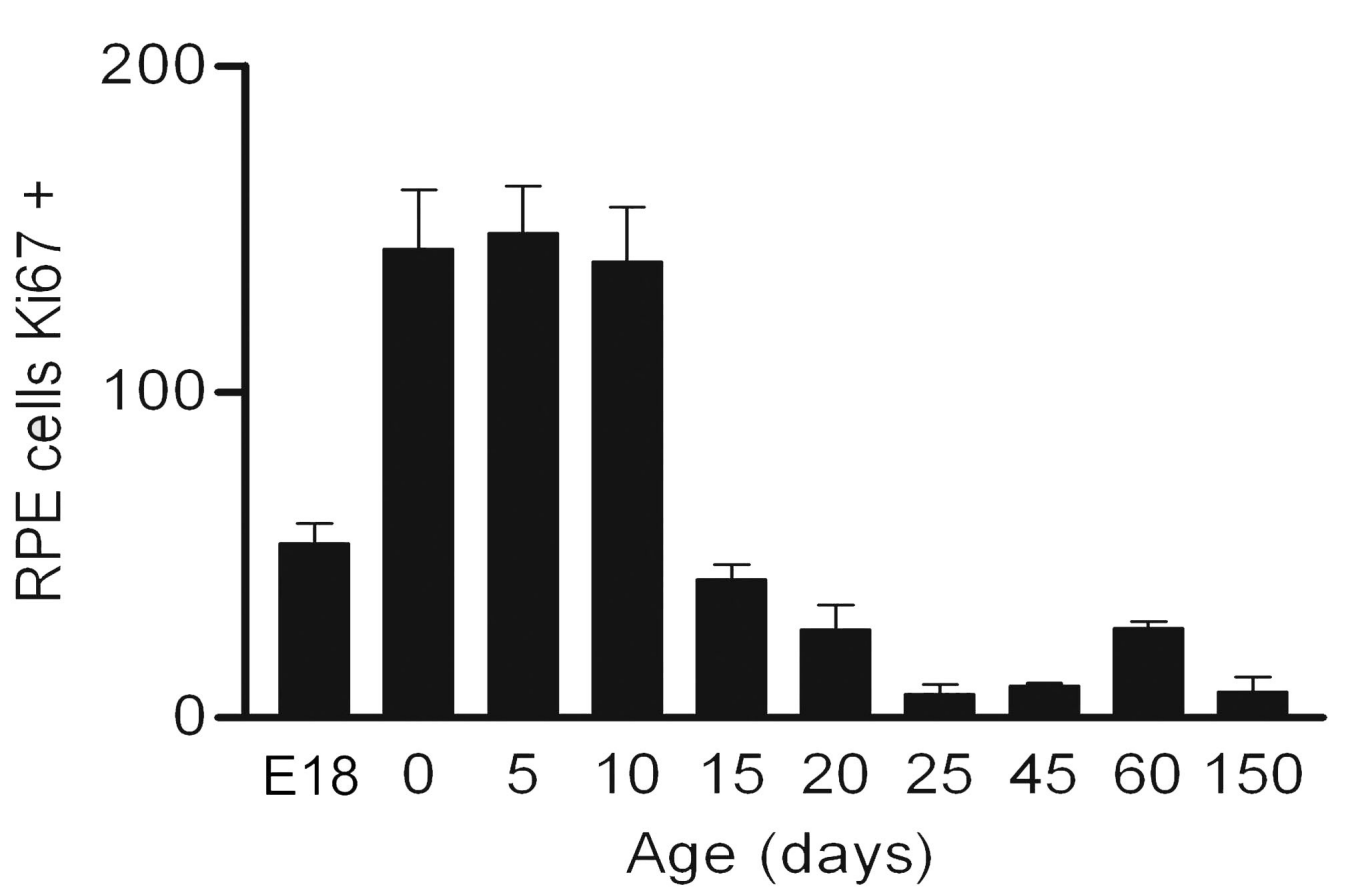
The number of Ki67-positive cells found in the RPE flat mounts of pigmented animals sampled from embryonic day 18 (E18) through to postnatal day 150. Relatively few cells were in the cell cycle at E18, which is when the normal patterns of cell division in developing tissue end. However there was a marked increase (ANOVA, p<0.0001) in the number of these cells on the day of birth (0), which was statistically significant compared to E18 (Newman-Keuls, p<0.001). From P0 on, cell number gradually declined with age.

As the retina develops with a center to periphery gradient, such that late cell division occurs in the peripheral retina [19], it is natural to ask whether the relatively peripheral patterns of Ki67 labeling found in the mature RPE are not simply a reflection of those patterns present during late development, or whether they represent a distinct and separate event. We labeled RPE flat mounts from pigmented animals at progressive stages from E18 through the postnatal period and into maturity with Ki67. Unfortunately, it was not possible to generate retinal whole-mounts of sufficient quality before E18 without inducing some damage to the periphery that resulted in loss of labeled cells. At E18, approximately 50 Ki67-positive cells could be identified in the RPE sheet. However, three days later on the day of birth, there was a large increase in the number of positive cells found, from around 50 to approximately 260, which is statistically significant compared to the earlier time point (Newman-Keuls, p<0.001; Figure 8). From this point onward the number of positive cells declined gradually until around p20–P25 when their number reached comparable levels to those found in older animals. At all of these stages of development, few if any Ki67-positive cells were identified in central regions, rather they were confined to equatorial and peripheral retinal regions similar to that shown in Figure 2 for the adult. These patterns of Ki67 labeling through postnatal development were similar in albinos, although the absolute number of positive cells found was markedly elevated (data not shown). These data are consistent with the notion that the increase in cell labeling found in the RPE around the time of birth may represent a distinct and separate event from earlier patterns that start centrally and end in the periphery.

## Discussion

Using three independent markers for proliferation and three independent functional markers of RPE cells, we have demonstrated that a proportion of mature RPE cells are retained in or have the capacity to enter into the cell cycle and divide in peripheral and equatorial retinal regions. The number of these cells were significantly elevated when pigment was absent. In pigmented animals multiple injections of BrdU only elevated cell numbers at 24 h intervals over 5 days, indicating that the cell cycle rate is probably slow. It is interesting in the light of this result that in an analysis of 50 of the most highly expressed retinal genes, 11 showed differential expression between central and peripheral human RPE. Of these, two were cell cycle genes that were downregulated in the central retina compared to the periphery [20].

Consideration of the relative distribution of binucleated cells and those in the cell cycle could lead to the conclusion that there might be a slow progressive wave of nuclear division across the retina, starting in the center and ending in the periphery. However, the presence of cells positive for BrdU that contained only one nucleus argues against this. Further, examination of data from younger and older animals does not support this assertion, as at every stage of development examined, the distribution of binucleated cells was largely confined to central and equatorial regions alone, and that of Ki67-positive cells to equatorial and peripheral regions (data not shown).

There are marked differences between pigmentation phenotypes in the number of Ki67 labeled cells found in their respective RPE. There are two possible reasons for this. First, during development albino retinas are more proliferative than pigmented because they lack dopa, an upstream element in the synthetic pathway of melanin, shown to encourage cell cycle exit [14,21]. It is possible that the absence or reduction of dopa during development has a persistent impact upon their retinas extending into maturity. Second, albinos have far fewer rod photoreceptors than normally pigmented animals [22,23], and hence the RPE phagocytotic workload is probably reduced. As RPE proliferation is enhanced by retinal detachment [8], it is possible that there is a relationship between photoreceptor numbers and their contact with the RPE, and the probability of RPE cells entering the cell cycle. However, this remains to be proven. There are likely to be significant differences between the rats used here other than their pigmentation phenotype, and include variation in genetic background and ocular light experience, among other difference. In light of this, an analysis of the number and distribution of Ki67-positive cells in mutant animals having photoreceptor dystrophies would be of value.

Why has cell division in the mature RPE not been identified before and why does it occur? Rodent RPE is rarely viewed in whole-mount because it is difficult to remove as a coherent tissue without becoming significantly damaged in the process. For this reason it is commonly viewed in section, where this important, but relatively sparse cell population would be hard to identify. Examination of normal adult retinas labeled with tritiated thymidine to mark dividing cells in cat failed to produce positive label in the RPE [8]. However, Ts’o et al. [13] noted two mitotic nuclei in mature albino retinas, but these could have been dividing nuclei as opposed to dividing cells, and were not noted in other retinas used in their study. Another reason these cells may not have been identified is simply that no investigation has used cell cycle markers, such as Ki67, on whole mount mature mammalian RPE before. In spite of this, a proliferative zone in the neural retina and the RPE at the retinal margin has been noted during early stages in the hatched chick [24,25], and here the level of proliferation is of sufficient magnitude to be obvious in section, but it is unclear how long this is sustained for.

While we demonstrate that a population of cells in the mature RPE divide, we have no data to indicate that the number of RPE cells actually increases with age. In fact in a recent study from this laboratory, a striking feature of rat RPE is that it shows little age-related cell loss (unpublished). Hence, if the peripheral RPE is undergoing gradual cell addition, then it is probable that this is a process that replenishes the tissue when it is subjected to normal age related cell loss. However, as RPE cell loss is likely to be pan retinal, the local addition of these cells toward the periphery would require gradual shifts in the RPE toward the central retina. Interestingly such a proposal has been made by Del Priore et al. [6]. They found evidence for cell death in the human RPE but no evidence for age-related changes in central cell density. Interestingly, the data on age related changes in the human RPE is mixed. Some studies find evidence for age-related loss [5], some no change [6], and another comprehensive analysis demonstrates an actual increase in density with age [26]. Whichever study is examined, there is a wide diversity in individual data. It is possible that a fundamental mechanism exists to replenish the RPE through peripheral division to balance cell loss, but that this balance is easily disrupted leading to significant variation between individuals.

The proliferative capacity of the RPE has an additional dimension in that it is enhanced if the retina is removed [8], although only in peripheral and not central regions [27]. Some amphibians have the ability to take this a stage further and are able to regenerate a new retina through transdifferentiation of the proliferating RPE population [9]. Mammals seem to have retained an element of this process in their ability to proliferate, but appear to lack the regulatory elements necessary to control RPE proliferation appropriately and induce transdifferentiation. However, if their entry into the cell cycle could be regulated, it would have very significant implications for disease processes where the RPE is either lost or damaged, such as age-related macular degeneration.

## References

[1] Curcio CA, Sloan KR, Kalina RE, Hendrickson AE (1990). Human photoreceptor topography.. J Comp Neurol.

[2] Cunea A, Jeffery G (2007). The ageing photoreceptor.. Vis Neurosci.

[3] Gao H, Hollyfield JG (1992). Aging of the human retina. Differential loss of neurons and retinal pigment epithelial cells.. Invest Ophthalmol Vis Sci.

[4] Balazsi AG, Rootman J, Drance SM, Schulzer M, Douglas GR (1984). The effect of age on the nerve fiber population of the human optic nerve.. Am J Ophthalmol.

[5] Panda-Jonas S, Jonas JB, Jakobczyk-Zmija M (1996). Retinal pigment epithelial cell count, distribution, and correlations in normal human eyes.. Am J Ophthalmol.

[6] Del Priore LV, Kuo YH, Tezel TH (2002). Age-related changes in human RPE cell density and apoptosis proportion in situ.. Invest Ophthalmol Vis Sci.

[7] Tropepe V, Hitoshi S, Sirard C, Mak TW, Rossant J, van der Kooy D (2001). Direct neural fate specification from embryonic stem cells: a primitive mammalian neural stem cell stage acquired through a default mechanism.. Neuron.

[8] Anderson DH, Stern WH, Fisher SK, Erickson PA, Borgula GA (1981). The onset of pigment epithelial proliferation after retinal detachment.. Invest Ophthalmol Vis Sci.

[9] Chiba C, Hoshino A, Nakamura K, Susaki K, Yamano Y, Kaneko Y, Kuwata O, Maruo F, Saito T (2006). Visual cycle protein RPE65 persists in new retinal cells during retinal regeneration of adult newt.. J Comp Neurol.

[10] Redmond TM, Yu S, Lee E, Bok D, Hamasaki D, Chen N, Goletz P, Ma JX, Crouch RK, Pfeifer K (1998). Rpe65 is necessary for production of 11-cis-vitamin A in the retinal visual cycle.. Nat Genet.

[11] Saari JC, Nawrot M, Kennedy BN, Garwin GG, Hurley JB, Huang J, Possin DE, Crabb JW (2001). Visual cycle impairment in cellular retinaldehyde binding protein (CRALBP) knockout mice results in delayed dark adaptation.. Neuron.

[12] Martinez-Morales JR, Rodrigo I, Bovolenta P (2004). Eye development: a view from the retina pigmented epithelium.. Bioessays.

[13] Ts'o MO, Friedman E (1967). The retinal pigment epithelium. I. Comparative histology.. Arch Ophthalmol.

[14] Ilia M, Jeffery G (1999). Retinal mitosis is regulated by dopa, a melanin precursor that may influence the time at which cells exit the cell cycle: analysis of patterns of cell production in pigmented and albino retinae.. J Comp Neurol.

[15] Kralj-Hans I, Tibber M, Jeffery G, Mobbs P (2006). Differential effect of dopamine on mitosis in early postnatal albino and pigmented rat retinae.. J Neurobiol.

[16] Cernak I, Stoica B, Byrnes KR, Di Giovanni S, Faden AI (2005). Role of the cell cycle in the pathobiology of central nervous system trauma.. Cell Cycle.

[17] Yang Y, Herrup K (2007). Cell division in the CNS: protective response or lethal event in post-mitotic neurons?. Biochim Biophys Acta.

[18] Stroeva OG, Mitashov VI (1983). Retinal pigment epithelium: proliferation and differentiation during development and regeneration.. Int Rev Cytol.

[19] Mann I. The development of the human eye. Grune & Stratton, New York: 1964.

[20] Ishibashi K, Tian J, Handa JT (2004). Similarity of mRNA phenotypes of morphologically normal macular and peripheral retinal pigment epithelial cells in older human eyes.. Invest Ophthalmol Vis Sci.

[21] Tibber MS, Whitmore AV, Jeffery G (2006). Cell division and cleavage orientation in the developing retina are regulated by L-DOPA.. J Comp Neurol.

[22] Donatien P, Aigner B, Jeffery G (2002). Variations in cell density in the ganglion cell layer of the retina as a function of ocular pigmentation.. Eur J Neurosci.

[23] Grant S, Patel NN, Philp AR, Grey CN, Lucas RD, Foster RG, Bowmaker JK, Jeffery G (2001). Rod photopigment deficits in albinos are specific to mammals and arise during retinal development.. Vis Neurosci.

[24] Fischer AJ, Reh TA (2000). Identification of a proliferating marginal zone of retinal progenitors in postnatal chickens.. Dev Biol.

[25] Fischer AJ (2005). Neural regeneration in the chick retina.. Prog Retin Eye Res.

[26] Harman AM, Fleming PA, Hoskins RV, Moore SR (1997). Development and aging of cell topography in the human retinal pigment epithelium.. Invest Ophthalmol Vis Sci.

[27] Kiilgaard JF, Prause JU, Prause M, Scherfig E, Nissen MH, la Cour M (2007). Subretinal posterior pole injury induces selective proliferation of RPE cells in the periphery in in vivo studies in pigs.. Invest Ophthalmol Vis Sci.

